# Celecoxib inhibits proliferation and survival of chronic myelogeous leukemia (CML) cells via AMPK-dependent regulation of β-catenin and mTORC1/2

**DOI:** 10.18632/oncotarget.13146

**Published:** 2016-11-07

**Authors:** Beatrice Riva, Marco De Dominici, Ilaria Gnemmi, Samanta A. Mariani, Alberto Minassi, Valentina Minieri, Paolo Salomoni, Pier Luigi Canonico, Armando A. Genazzani, Bruno Calabretta, Fabrizio Condorelli

**Affiliations:** ^1^ Department of Pharmacological Sciences, Università del Piemonte Orientale “A. Avogadro”, 28100 Novara, Italy; ^2^ Sidney Kimmel Cancer Center, Thomas Jefferson University, Philadelphia 19107, PA, USA; ^3^ MRC Centre for Inflammation Research, University of Edinburgh, Edinburgh EH16 4TJ, UK; ^4^ Samantha Dickson Brain Cancer Unit, University College London Cancer Institute, London WC1E 6BT, UK

**Keywords:** celecoxib, chronic myelogenous leukemia, cyclooxygenase-2, beta-catenin, AMP-activated kinase

## Abstract

CML is effectively treated with tyrosine kinase inhibitors (TKIs). However, the efficacy of these drugs is confined to the chronic phase of the disease and development of resistance to TKIs remains a pressing issue. The anti-inflammatory COX2 inhibitor celecoxib has been utilized as anti-tumour drug due to its anti-proliferative activity. However, its effects in hematological malignancies, in particular CML, have not been investigated yet. Thus, we tested biological effects and mechanisms of action of celecoxib in Philadelphia-positive (Ph^+^) CML and ALL cells.

We show here that celecoxib suppresses the growth of Ph^+^ cell lines by increasing G1-phase and apoptotic cells and reducing S- and G2-phase cells. These effects were independent of COX2 inhibition but required the rapid activation of AMP-activated protein kinase (AMPK) and the consequent inhibition mTORC1 and 2. Treatment with celecoxib also restored GSK3β function and led to down-regulation of β-catenin activity through transcriptional and post-translational mechanisms, two effects likely to contribute to Ph^+^ cell growth suppression by celecoxib.

Celecoxib inhibited colony formation of TKI-resistant Ph^+^ cell lines including those with the T315I BCR-ABL mutation and acted synergistically with imatinib in suppressing colony formation of TKI-sensitive Ph^+^ cell lines. Finally, it suppressed colony formation of CD34^+^ cells from CML patients, while sparing most CD34^+^ progenitors from healthy donors, and induced apoptosis of primary Ph^+^ ALL cells.

Together, these findings indicate that celecoxib may serve as a COX2-independent lead compound to simultaneously target the mTOR and β-catenin pathways, key players in the resistance of CML stem cells to TKIs.

## INTRODUCTION

Celecoxib is a sulfonamide COX2 inhibitor (COXib) used in the therapy of osteoarthritis and rheumatoid arthritis. In addition to its anti-inflammatory activity, celecoxib exerts anti-proliferative effects on transformed cells, as shown in some solid tumors. In particular, it is the only COXib used for the therapy of Familial Adenomatous Polyposis (FAP) patients with the goal to prevent its evolution toward colon cancer [1-3] by inhibiting the COX2-dependent secretion of prostaglandin E2 by adenomatous cells.

Nevertheless, several reports indicate that celecoxib exerts cell-autonomous anti-proliferative and pro-apoptotic effects also in cancer cell lines that do not express COX2 [4,5]. In line with these findings, dimethyl-celecoxib, a close structural analogue of celecoxib that lacks anti-COX2 activity (“non-COXib”), mimics the anti-tumor effects of celecoxib [6-8].

Alternative targets of celecoxib are at present elusive although recent reports have focused on PDK1, SERCA, carbonic anhydrase, NFκB, and survivin [9,10], all of which are typically inhibited at concentrations higher than those usually required for COX2 inhibition [8,11,12].

Chronic myelogenous leukemia (CML), a myeloproliferative disorder caused by the BCR-ABL1 oncoprotein, is an ideal model to dissect COX2-dependent and COX2-independent mechanisms of celecoxib growth inhibition because the role of many signal transduction pathways in CML cell proliferation and survival is well established, allowing the effects of celecoxib to be linked to the modulation of specific BCR-ABL-regulated pathways. Moreover, drugs not previously known to play a role in CML, such as the anti-diabetic drug pioglitazone, appear to have important and unexpected effects in CML [13], raising the possibility that growth-inhibitory effects of celecoxib in Ph^+^ cells may be therapeutically relevant.

In CML, the BCR-ABL chimeric oncoprotein which functions as a constitutively active tyrosine kinase is also necessary for disease maintenance; thus, CML provides an ideal model for testing the effects of “targeted therapies”. Indeed, treatment with imatinib or second-generation tyrosine-kinase inhibitors (TKIs) has markedly improved the survival of CML patients; however, individual intolerance to these inhibitors, the emergence of clones with TKI-resistant BCR-ABL mutations, and the observation that leukemia-initiating/stem cells are intrinsically resistant to these drugs, in part due to overacting PI3K/AKT/mTOR and β-catenin pathways, support the ongoing search for new drugs targeting CML stem cells [14,15].

We show here that celecoxib, at concentrations near those required for its anti-inflammatory effects, suppresses proliferation and colony formation of imatinib-sensitive and resistant Ph^+^ cell lines and primary cells, including CD34^+^ CML cells. Of greater importance, celecoxib had only modest effects on colony formation of normal CD34^+^ progenitors. Mechanistically, the effects of celecoxib appear to be COX2-independent through AMP-dependent kinase regulation of mTOR and β-catenin, two important mediators of TKI resistance in CML stem cells.

## RESULTS

### Celecoxib impairs proliferation and induces cell death of CML cell lines in a COX2-independent manner

**Figure 1:**
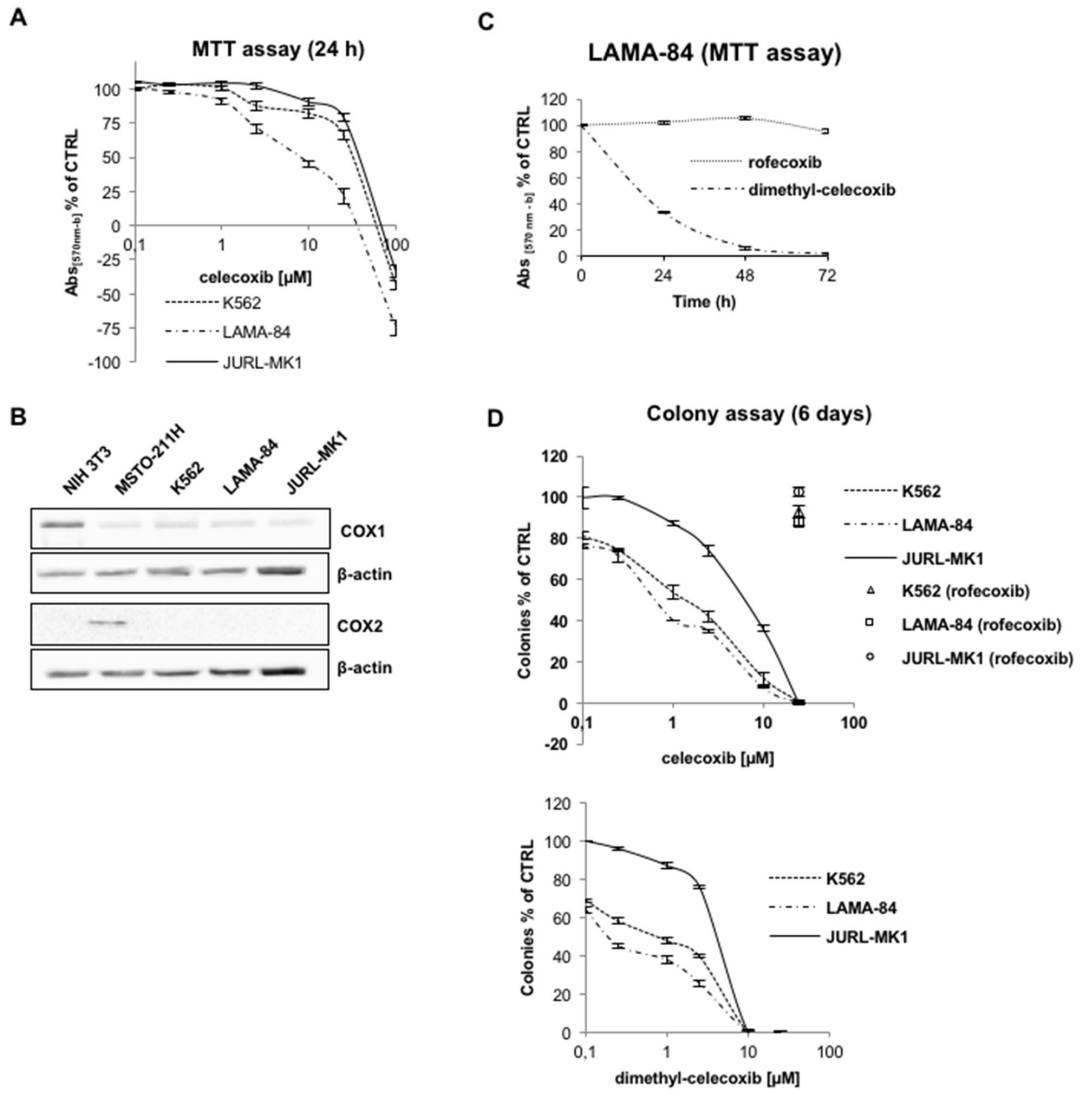
Effects of celecoxib on CML cell proliferation **A.** Cell viability of CML cell lines treated for 24 hours with increasing concentrations of celecoxib. According to NCI screening methodology (https://dtp.cancer.gov/discovery_development/nci-60/methodology.htm), the discrimination between cytostatic and cytotoxic effects in the upper diagram is evidenced by expressing the percentage of growth inhibition as [(Ti-Tz)/(C-Tz)] x 100, for concentrations for which Ti>/=Tz, or [(Ti-Tz)/Tz] x 100, for concentrations for which Ti<Tz (Tz: time zero; Ti: test growth after 24 hours in presence of the drug; C: control growth after 24 hours in presence of 0.1% DMSO). Values are means from three independent experiments ± S.E.M. **B.** Immunoblots of cyclooxygenase-1 (**COX1**) and -2 (**COX2**) in CML cell lines. β-actin levels were used to assess equivalence of protein loading. NIH-3T3 and MSTO-211H protein lysates were used as positive controls respectively for COX1 and COX2 expression. **C.** Time-course of LAMA-84 cells viability after treatments with 25 μM rofecoxib or dimethyl-celecoxib. Values are means from three independent experiments ± S.E.M. **D.** Dose-response curves of celecoxib (top) and dimethyl-celecoxib (bottom) in colony formation assays of CML cells lines. Cells (1,250/well) were grown for 6 days on 80% methylcellulose culture media after treatment with drugs. Open labels indicate treatment with rofecoxib. Ctrl cells were treated with equal amounts of DMSO (0.1%). Values represent the mean of three independent experiments in duplicate ±S.E.M.

The effect of celecoxib on CML viability was assessed in three Ph^+^ CML-blast crisis cell lines (K562, LAMA-84, JURL-MK1) treated for 24 hours (Figure 1A). Inhibition of proliferation was especially evident in the LAMA-84 cell line (EC50 of approximately 23.8 μM), while the JURL-MK1 cell line was the least responsive (EC50 of 75.2 μM). Longer exposures to celecoxib led to a decrease in EC50s (e.g. for 6 days 8.2 μM for LAMA-84 and 23.8 μM for JURL-MK1; Supplementary Figure S1). Then, we assessed whether the anti-proliferative effect of celecoxib was due to COX2 inhibition. Immunoblots of cell lysates revealed that the three cell lines express very low levels of COX1, which is not targeted by celecoxib, whereas expression of COX2 was not detectable (Figure 1B). We also assessed the anti-proliferative effects of dimethyl-celecoxib, an analogue unable to inhibit COX2 [11] and of rofecoxib, a structurally distinct COX2 inhibitor. Dimethyl-celecoxib exhibited an EC50 of 16.8 μM in LAMA-84 at 24 hours. By contrast, rofecoxib had no effect, even at the highest concentration (25 μM) tested for 72 hours (Figure 1C).

The therapeutic potential of putative anti-leukemia drugs is best measured by evaluating how efficiently they suppress colony formation in a semisolid medium. When cells were grown in culture media containing 80% methylcellulose, celecoxib or dimethyl-celecoxib suppressed colony formation in a concentration-dependent manner (Figure 1D) and, as expected, their activity was not due to inhibition of COX2 since rofecoxib had no effect. Again, LAMA-84 cells were the most sensitive to treatment with celecoxib or dimethyl-celecoxib; however, the EC50 of celecoxib calculated on the basis of the methylcellulose assay (0.5 μM; Figure 1D) was markedly lower than that based on the MTT assays (8.2 μM, after 6 days; Supplementary Figure S1).

As shown in Figure 1A, celecoxib, at concentrations lower than 25 μM, has a cytostatic rather than a cytotoxic effect, as MTT absorbance at 24 hours is not inferior to that at the zero time-point. This was confirmed when cell counts were used as a direct measure (data not shown).

**Figure 2:**
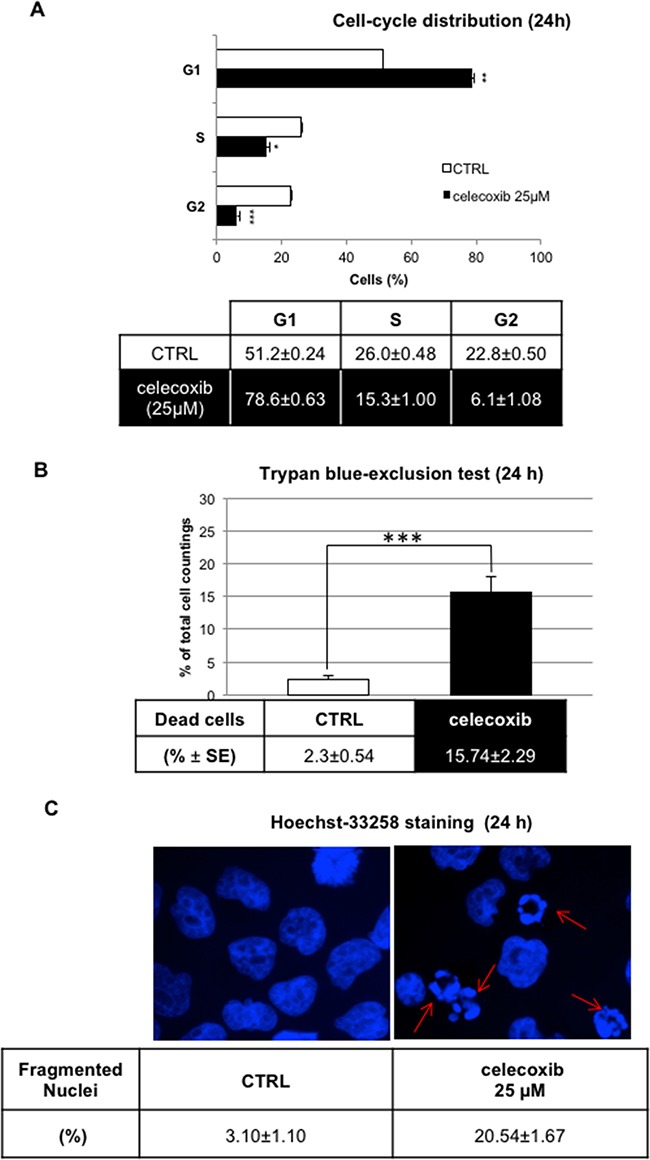
Effect of celecoxib on Ph^+^ cell viability by DNA content analysis **A.** LAMA-84 cell-cycle distribution assessed by flow cytometry after propidium iodide staining. Results (means ± S.E.M of n=3 samples; *P ≤ 0.05, **P ≤ 0.01, ***P ≤ 0.001) depict a representative experiment of three experiments that yielded overlapping results. **B.** Trypan-blue exclusion test on LAMA-84 cells treated with celecoxib (25 μM) for 24 hours.A minimum of 100 cells was scored for each condition. Data on table represent the means of three independent experiments ± S.E.M. ***P ≤ 0.001 **C.** UV-fluorescence(60 x magnification) of Hoechst33258-stained LAMA-84 cell nuclei after 24-hours treatment. Arrows indicate the fragmented nuclei of apoptotic cells. Data represent mean percentages of apoptotic cells from three independent experiments ± S.E.M.

Single cell DNA content analysis of propidium iodide-stained nuclei showed that celecoxib (25 μM) caused a marked increase in the G1 fraction (78.5 % vs. 51.2 % of controls) of LAMA-84 cells (Figure 2A) paralleled by a marked decrease of S (15.4 % vs 26.0 % of controls) and G2 phase cells (6.1 % vs 22.8 %). Yet, trypan blue exclusion data (Figure 2B) and microscopic counting of fragmented nuclei stained with Hoechst-33258 (Figure 2C) revealed that treatment with 25 μM celecoxib caused also the death of about 15-20 % of the cells.

### Celecoxib impairs the activity of the β-catenin/Tcf/Lef axis

**Figure 3:**
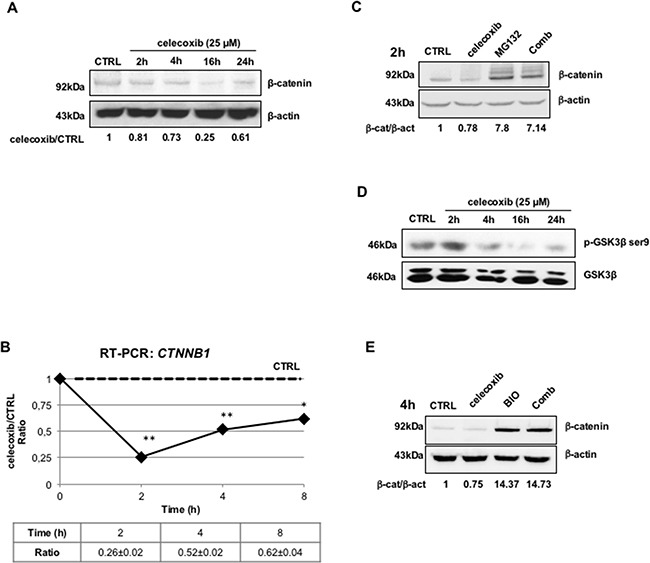
Celecoxib causes β-catenin down-regulation in a GSK3β- and proteasome-dependent manner **A.** β-catenin protein expression in LAMA-84 cells exposed to celecoxib (25 μM) at different time-points. Numbers underneath lanes represent the treated/control ratio of the optical densities of β-catenin immuno-reactive bands. To exclude the influence of spontaneous fluctuations in β-catenin expression, at each time-point, protein extracts of drug-treated cells were compared with the respective controls. Levels of β-actin were also calculated and used to normalize protein loadings. **B.** Expression of β-catenin gene (*****CTNNB1*****) transcripts in LAMA-84 cells treated with celecoxib (25 μM). The levels of *CTNNB1* mRNAs were normalized using the expression of GAPDH transcripts as reference. Ratios represent means of three independent experiments ± S.E.M. *P ≤ 0.05, **P ≤ 0.01 **C.** Immunoblots showing that pharmacological inhibition of the proteasome reverts the effects of celecoxib on β-catenin protein degradation. LAMA-84 cells were treated for 2 hours (2h) with celecoxib (25 μM), MG132 (20 μM), or in combination (**Comb**). Values displayed underneath lanes represent ratios of the optical densities of β-catenin (β-cat) immuno-reactive bands over those of β-actin (β-act). **D.** Immunoblots showing that celecoxib causes a time-dependent decrease of GSK3β phosphorylation in LAMA-84 cells. Levels of total GSK3β protein are displayed to account for protein loading variations. **E.** Immunoblot showing that GSK3β inhibition with BIO antagonizes celecoxib-induced down-regulation of β-catenin. LAMA-84 cells were treated for 4 hours with 25 μM celecoxib, 10 μM BIO, or a combination of both (**Comb**). As for Figure 4A, values underneath lanes represent ratios between the optical densities of β-catenin and β-actin immuno-reactive bands (**β-cat/β-act**).

A great deal of attention has recently been paid to the role of signal transduction pathways involved in maintaining the self-renewal of leukemic cells. CML stem cells appear to rely, among others, on the β-catenin/Tcf-Lef-dependent transcription program [16]. This pathway is aberrantly activated in CML mononuclear cells both in the chronic phase and the blast crisis stage [17]. In particular, BCR-ABL can inhibit glycogen synthase kinase 3-Beta (GSK3β) that regulates ubiquitination and proteasome-dependent degradation of β-catenin; in addition, BCR-ABL may directly activate the transcriptional function of β-catenin by phosphorylating it on tyrosine residues [18,19]. Thus, we tested the hypothesis that celecoxib may exert its effect on CML cells through negative modulation of β-catenin. Indeed, celecoxib (25 μM) caused a marked decrease of β-catenin protein expression in LAMA-84 cells treated for 2-24 hours. This effect was already visible after 2 hours and peaked after a 16-hours treatment (Figure 3A). We next assessed whether the effect was due to transcriptional regulation of β-catenin expression. Indeed, treatment with celecoxib for 2 hours led to a marked decrease (almost by 75%) of β-catenin mRNA levels as measured by RT-PCR, an effect that lasted for 24 hours after a single drug treatment (Figure 3B).

We also assessed whether inhibition of proteasome activity by treatment with MG132 could restore the effect of celecoxib on β-catenin expression. Cells were exposed to celecoxib only for 2 hours in order to limit its transcriptional effects, although still ensuring a 20% reduction of β-catenin levels. As shown in Figure 3C, MG132 treatment alone caused a seven-fold increase of β-catenin protein levels in comparison to control treatment (0.1% DMSO) but, more importantly, this effect was also detected in cells treated with celecoxib.

A pre-condition for the proteasome-dependent degradation of β-catenin is its N-terminal phosphorylation by GSK3β. To monitor the activity of GSK3β in celecoxib-treated cells, we measured the levels of ser-9 phosphorylation, which denotes the inactive form of the kinase [20]. As shown in Figure 3D, celecoxib inhibited the phosphorylation of this kinase, already after a 4-h treatment. Further proof that GSK3β reactivation played a role in the celecoxib-induced proteasomal degradation of β-catenin was obtained upon treatment with the GSK3β inhibitor BIO (10 μM); exposure to this inhibitor caused, on its own, a fourteen-fold increase of β-catenin levels as compared to mock treatment (0.1% DMSO), an effect which was not blocked by simultaneous administration of celecoxib (Figure 3E).

**Figure 4:**
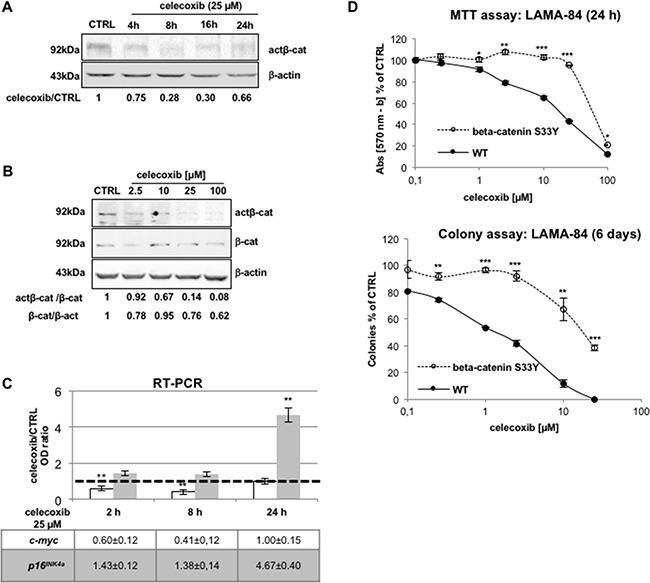
Negative modulation of the Tcf/Lef/β-catenin pathway by celecoxib is essential for its biological effects **A, B.** Celecoxib reduces the levels of the transcriptional-active form of β-catenin in a time- (**A**) and concentration-dependent manner (**B**) in LAMA-84 cells. In A, optical densities of act-β-cat immuno-reactive bands were normalised by the intensity of β-actin in order to calculate treatment-to-control (**celecoxib/CTRL**) ratios displayed underneath lanes. In B, optical densities of act-β-cat, total β-cat, and β-actin immuno-reactive bands (**β-act**, used to to normalize protein loadings between lanes) were assessed to calculate the **act-β-cat/β-cat** and **β-cat/β-act** ratios displayed underneath lanes. **C.** Celecoxib affects the transcription of β-catenin-target genes, *c-myc* and *p16^INK4a^* in LAMA-84 cells as measured by RT-PCR. Data were normalized using GAPDH transcripts as reference. The dotted line intercepting the vertical axis at the unit indicates mRNA expression in 0.1 % DMSO-treated cells (**CTRL**). Values represent means of three independent experiments ± S.E.M. **P ≤ 0.01. **D.** Effect of celecoxib in LAMA-84 cells expressing wild-type β-catenin (**WT**) or its serine-to-tyrosine 33 mutant (**beta-catenin S33Y**) assessed via the MTT assay (viability; top) or the colony assay (clonogenicity; bottom). Data represent averages of three independent experiments made in duplicate ± S.E.M. *P ≤ 0.05, **P ≤ 0.01, ***P ≤ 0.001.

Previous studies have shown that cytosolic stabilization of unphosphorylated β-catenin allows its translocation to the nucleus [21,22]. Thus, immunoblotting of lysates of LAMA-84 cells with an antibody recognizing only the unphosphorylated form of β-catenin (actβ-cat), revealed that celecoxib reduced the levels of this transcription-competent isoform in a time- and concentration-dependent manner (Figure 4A and 4B, respectively).

The β-catenin/Tcf/Lef complex *trans*-activates genes with growth-promoting or anti-apoptotic activity. One of such target genes is the *c-myc* oncogene; thus, quantitative RT-PCR analysis of *c-myc mRNA* levels in LAMA-84 cells treated with 25 μM celecoxib (for 2, 8 or 24 hours) revealed a marked decrease (more evident after 8 hours) in the levels of *c-myc* transcripts (Figure 4C) and protein levels (Supplementary Figure S2), consistent with inhibition of active β-catenin.

Nuclear accumulation of β-catenin impairs the transcription of the *CDKN2A* gene, which encodes the p16^INK4a^ tumour suppressor protein, with an inhibitory effect on cell cycle progression [23,24]. RT-PCR of p16^INK4a^ mRNA levels assessed after treatment with 25 μM celecoxib revealed a significant increase of these transcripts, although only after a 24-h treatment (Figure 4C).

To establish a correlation between the effect of celecoxib on β-catenin protein stability and on proliferation/colony formation of Ph^+^ CML cells, we generated a LAMA-84 parental cell line expressing a constitutively active mutant form of β-catenin (β-catenin S33Y) that cannot be targeted to the proteasome because it is not phosphorylated by GSK3β [25,26]. As expected, cells expressing the degradation-resistant form of β-catenin were significantly more resistant than parental cells to either acute (Figure 4D, upper panel) or chronic (Figure 4D, lower panel) exposure to celecoxib.

### Celecoxib inhibits the activity of mammalian target of rapamycin complex 1 (mTORC1) and 2 (mTORC2)

Since GSK3β causes the disassembly of mTORC1 [27], we next investigated the effect of celecoxib on mTOR and its downstream targets.

**Figure 5:**
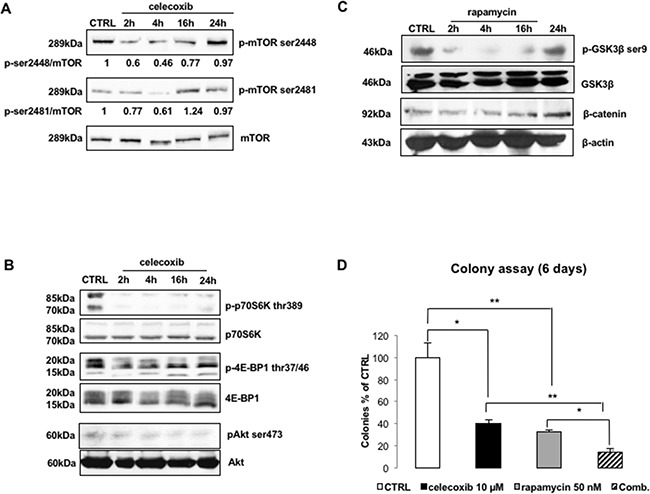
Celecoxib modulates the activity of mTOR kinase **A.** Immunoblots of LAMA-84 lysates treated with celecoxib (25 μM) to explore mTOR phosphorylation. Values underneath lanes represent the optical densities of p-mTOR immuno-reactive bands corrected by the total levels of mTOR. **B.** Immunoblots of LAMA-84 lysates treated with celecoxib (25 μM) to explore phosphorylation/activation of mTORC1 and mTORC2 down-stream targets (p-p70S6K thr-389, p-4E-BP1 thr-37/46, pAkt ser-473). Levels of total p70S6K, 4E-BP1 and Akt are displayed to compare protein loadings between lanes. **C.** Time-course of GSK3β phosphorylation (**p-GSK3β**-**ser9**) and β-catenin (**β-cat**) protein expression following to inhibition of mTORC1 complex in LAMA-84 cells treated with 50 nM rapamycin. Levels of β-actin (**β-act**) are displayed as proof of equal loading between lanes. **D.** mTORC1 inhibition and CML cell clonogenicity. LAMA-84 cells were exposed to 50 nM of rapamycin, alone or in combination (**Comb**) with 10 μM celecoxib. Results are expressed as percentages of colonies counted, after 6 days, in drug-treated groups as compared to controls. Data represent averages of three independent experiments made in duplicate. *P ≤ 0.05, **P ≤ 0.01.

Figure 5A shows that celecoxib induced a time-dependent decrease of ser-2448 phosphorylation, an effect that was maximal within 4 hours of treatment. Surprisingly, phosphorylation of mTOR on ser-2481 was also diminished, although transiently, suggesting that, in contrast to rapamycin and its congeners, celecoxib exerts its inhibitory activity on both mTORC1 and mTORC2 complexes [28,29].

In agreement with these findings, treatment with celecoxib reduced the phosphorylation of S6 Kinase (p70-S6K) and eIF4E binding protein (4E-BP1), the two most important mTORC1 targets, as demonstrated by use of phospho-specific antibodies directed against thr-389 of p70S6K and the N-terminal domain of 4E-BP1 (Figure 5B). Similarly, celecoxib caused also a decrease in the phosphorylation of ser-473 Akt, which is the immediate target of mTORC2 (Figure 5B).

Since p70-S6K can negatively modulate GSK3β via phosphorylation of ser-9, it is possible that celecoxib-induced re-activation of GSK3β might be either the reason and/or the consequence of an impairment of mTORC1 function. Therefore, we used rapamycin (50 nM) to block mTORC1 activity and assessed whether this treatment recapitulates the effects induced by celecoxib. Surprisingly, although treatment with 50 nM rapamycin was as effective as 25 μM celecoxib in causing a time-dependent reduction of GSK3β phosphorylation on ser-9 (with a peak after 4 hours of treatment), it did not modify the levels of β-catenin (Figure 5C). On the other hand, colony formation assays of LAMA-84 cells treated with rapamycin (50 nM) and/or celecoxib (10 μM) yielded similar inhibition of GSK3β phosphorylation while the combination was much more potent (86.04±3.45% reduction vs control) than either drug used alone (celecoxib: 59.98±3.61%; rapamycin: 67.74±2.04%) (Figure 5D).

### The AMP-activated protein kinase (AMPK) contributes to the effects of celecoxib through a Ca^2+^-dependent mechanism

The re-activation of GSK3β induced in CML cells by both celecoxib and rapamycin suggested that it might be the consequence of mTORC1 inhibition rather than its cause. Moreover, the inability of rapamycin to entirely recapitulate the effects of celecoxib suggested that the molecular mechanisms linking treatment with celecoxib to impaired mTORC1 activity remain unclear.

It is known that AMPK activity precedes that of GSK3β in the inhibition of mTORC1 complex, thus placing this complex under the control of both metabolic stress and growth factors [30-32]. With this in mind, we tested whether celecoxib inhibited mTORC1, primarily through the activation of AMPK, an event that may be reinforced by re-activation of GSK3β.

**Figure 6:**
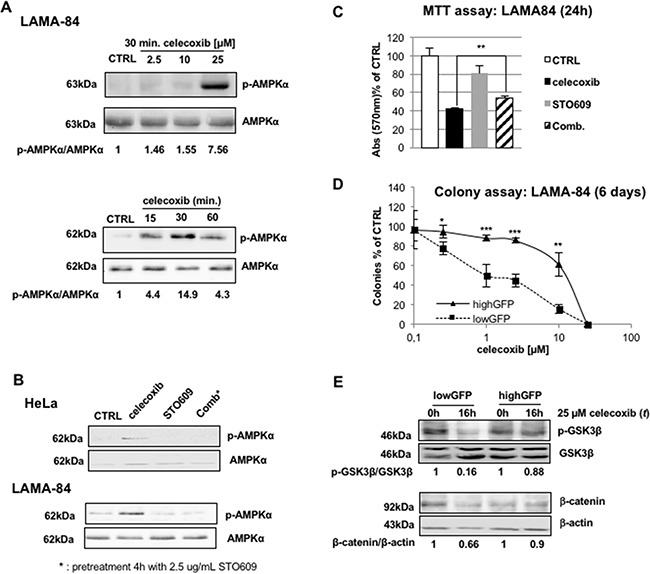
AMP-dependent kinase (AMPK) is activated by celecoxib in a Ca^2+^-dependent, AMP-independent, manner **A.** Concentration-response curve of celecoxib (top) and time-course using 25 μM celecoxib (bottom) of activation/phosphorylation of AMPK in LAMA-84 cells. Protein lysates were probed with antibodies that recognize the α-subunit of AMPK (**AMPK**) or its active form phosphorylated on thr-172 (**p-AMPKα**). Values underneath lanes express the ratio of the optical densities of p-AMPKα over total AMPK immuno-reactive bands. **B.** Celecoxib-induced activation of AMPK relies on Ca^2+^/calmodulin kinase kinase (CaMKK) function. The LKB1-deficient human cell line HeLa (upper panel) or LAMA-84 (bottom) were treated with 25 μM celecoxib (for 30 minutes), the CaMKK inhibitor STO609 (2.5 μg/mL, 4 hours), or a combination of both (**Comb**). Protein lysates were probed with AMPK or p-AMPKα specific antibodies. **C.** Inhibition of CaMKK limits the effect of celecoxib on CML blasts viability. LAMA-84 cells were treated for 24 hours with 25 μM celecoxib, 2.5 μg/ml STO609, or both, as described in Figure 6B. Histograms describe percent reductions of MTT values. Values are mean of three independent experiments ± S.E.M. **P ≤ 0.01. **D.** Dose-response curves of the colony assay of lowGFP (basal expression of AMPKα) and highGFP (low expression of AMPKα) LAMA-84 cells exposed to celecoxib. Cell colonies were scored 6 days after treatment and seeding. Values represent the average of three independent experiments in duplicate, ± S.E.M. *P ≤ 0.05, **P ≤ 0.01, ***P ≤ 0.001 **E.** GSK3β phosphorylation (**p-GSK3β**; top) and β-catenin (bottom) protein levels in lowGFP (basal expression of AMPKα) and highGFP (low expression of AMPKα) LAMA-84 cells. Ratios between the optical densities of p-GSK3β and GSK3β immuno-reactive bands are displayed underneath lanes of the top panel. Values displayed underneath lanes of the bottom panel are the ratio of the optical densities of the β-catenin immuno-reactive band over that of β-actin.

As shown in Figure 6A, celecoxib treatment increased AMPKα thr-172 phosphorylation, the active form of the catalytic subunit, in a concentration-dependent manner (upper panel) as early as 15 min after treatment.

AMPK is switched-on not only in response to a metabolic demand, represented by a relative increase of AMP over ATP concentration, but also after an increase of intracellular calcium, through the Ca^2+^/calmodulin-dependent kinase kinase (CaMKK) [29,32]. Thus, we investigated which of the two pathways mediates the effect of celecoxib on AMPK using the HeLa human cell line that lacks expression of LKB1, the kinase responsible of AMP-dependent activation of AMPKα [31]. As shown in Figure 6B, phosphorylation of AMPKα on thr-172 was induced after treatment with celecoxib, suggesting that LKB1 is dispensable for this effect of the drug. Conversely, co-administration of the CaMKK specific inhibitor, STO-609 (2.5 μg/mL), completely abolished the phosphorylation of AMPKα (Figure 6B). These effects were also observed in LAMA-84 cells (Figure 6B).

The involvement of CAMKK-induced activation of AMPK in mediating the effect of celecoxib was further supported by MTT assays, where STO609, albeit having some intrinsic activity, partially reversed the effect of celecoxib (Figure 6C).

To provide conclusive proof for the role played by AMPK in the anti-leukemic effect of celecoxib, we reduced the expression of its α- catalytic subunit generating LAMA-84 cells with low (highGFP; approximately 63% reduction; Supplementary Figure S3) or normal (lowGFP) levels of AMPKα using RNAi (see methods). Colony assays showed that decreased expression of this kinase was associated with decreased sensitivity to celecoxib treatment (Figure 6D). Moreover, in highGFP cells (with low levels of AMPKα), celecoxib failed to down-regulate both GSK3β and β-catenin expression (Figure 6E), indicating that activation of AMPK is essential for the effects of celecoxib. Surprisingly, OSU-03012, a non-COXib structural analogue of celecoxib that is in clinical development for the treatment of lymphoma patients and has been described as PDK1 inhibitor [33,34], reproduced the effects of celecoxib (Supplementary Figure S4), suggesting that activation of AMPK might be also relevant for the effects of this compound.

In support of the central role of AMPK activation for the effects of celecoxib, metformin, a first-line medication in the treatment of type II diabetes known to activate AMPK [35], acted similarly to celecoxib in suppressing CML cells growth (MTT, colony assays and cell cycle profiles are displayed in Supplementary Figure S5).

### Effects of celecoxib, alone or with imatinib, on colony formation of Ph^+^ cell lines (TKI-sensitive or resistant) and primary CD34^+^ CML and acute lymphoblastic leukemia (ALL) cells

**Figure 7:**
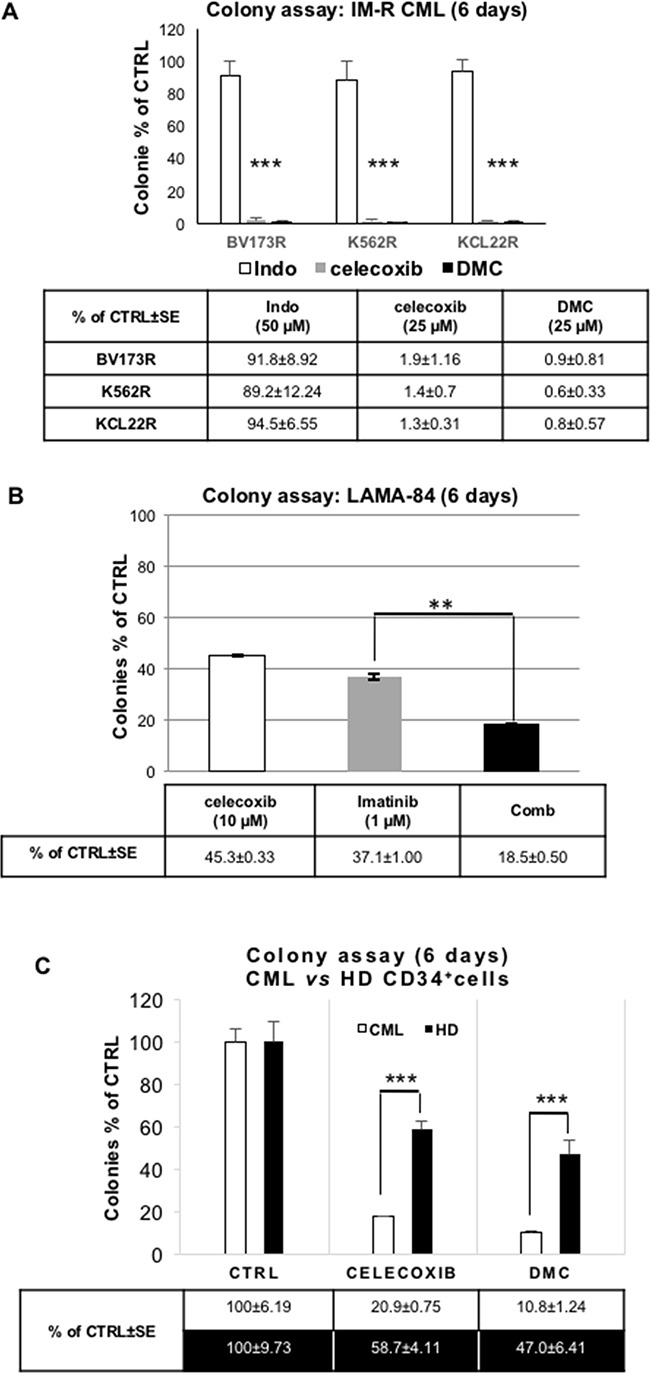
Effect of celecoxib, dimethyl-celecoxib, or the COX1/COX2 inhibitor indomethacin on colony formation of Ph^+^ CML cells and normal CD34^+^ progenitors **A.** Colony assay of imatinib-resistant (**IM-R**) CML blasts (cell lines: **BV173R**, **K562R**, **KCL22R**) treated with celecoxib (25 μM) or dimethyl-celecoxib (25 μM; **DMC**). Indomethacin (Indo; 50 μM), a COX1 and COX2 inhibitor, was included as a control. Values are the means of three independent experiments made in duplicate ± S.E.M. ***P ≤ 0.001. **B.** Colony assay of LAMA-84 cells treated with celecoxib (10 μM) and/or imatinib (1 μM). Values are the means of three independent experiments in duplicate ± S.E.M. **P ≤ 0.01. **C.** Colony assay of celecoxib or dimethyl-celecoxib (DMC) treated CD34^+^ CML cells (mean of 4 different samples) or normal cord blood-derived CD34^+^ cells from healthy donors (HD; mean of 4 different samples). Cells were treated with 25 μM of either drug before seeding (10,000 cells/plate) onto 80% methylcellulose in the presence of a cytokine cocktail. Colonies were scored after 9 days; ***P ≤ 0. 001.

TKIs are the cornerstones of current CML therapy, although resistance to these drugs is frequently observed. Thus, we tested the effect of celecoxib in imatinib-resistant CML-blast crisis cells. As shown in Figure 7A, 25 μM celecoxib or dimethyl-celecoxib, but not indomethacin (a COX1 and COX2 inhibitor), completely eradicated colony formation of three Ph^+^ TKI-resistant cell lines (BV173R, K562R, KCL22R; for details see the paragraph dedicated to *in vitro* cell lines in “materials and methods”).

We also tested whether imatinib and celecoxib have additive effects on the clonogenicity of TKI-sensitive LAMA-84 CML-blast crisis cells. As shown in Figure 7B, celecoxib, although used at a sub-optimal concentration (10 μM), potentiated the inhibitory effect of a therapeutic dose of imatinib (1 μM). Importantly, the Combination Index (CI) of the celecoxib/imatinib co-treatment, calculated on the inhibition of colony formation obtained by crossing four different concentrations of the two drugs (corresponding, respectively, to their EC20, 40, 60, 80), was less than one, indicating that this drug-drug interaction is synergistic according to Loewe's rule [36].

To determine whether celecoxib affects also the proliferation and survival of primary CML blasts, we performed colony assays of CD34^+^ cells from four chronic-phase CML patients. Strikingly, treatment with 25 μM of either celecoxib or dimethyl-celecoxib suppressed almost entirely CML cell colony formation (Figure 7C, white columns). By contrast, these treatments spared a sizable number of colonies derived from normal CD34^+^ progenitors (Figure 7C, black columns), demonstrating a relative selectivity of celecoxib and dimethyl-celecoxib toward BCR-ABL-expressing cells.

Finally, treatment with celecoxib or its dimethyl derivative suppressed the proliferation of Ph^+^ blasts isolated from ALL patients, including one resistant to imatinib and ponatinib, as assessed by means of cell counting (Supplementary Figure S6). Of note, this effect was associated with a significant increase of apoptosis, as indicated by detection of caspase 3 activity (Supplementary Figure S6).

## DISCUSSION

The evidence that TKI-based therapies are unable to eradicate CML stem cells supports the ongoing search for novel treatments that, by targeting these cells, may prevent disease relapse or transition to blast crisis [37]. A recent work focusing on the use of the anti-diabetic drug pioglitazone [13] has demonstrated that such treatments may be identified among those originally validated for diverse applications. A drug that may have similar behaviour is celecoxib since, in addition to its COX2-dependent anti-inflammatory activity, it is the only COXib that suppresses proliferation of cancer cells through unclear mechanisms. Indeed, celecoxib induces similar growth-inhibitory effects in COX2^(+/+)^, COX2^(+/-)^ and COX2^(-/-)^ mouse embryo fibroblasts, as well as in COX2-negative hematopoietic cell lines, including the K562 line [38-40], raising the question of which ones are the relevant targets of celecoxib in cancer cells.

We show here that the growth suppression of Ph^+^ CML and ALL cells by celecoxib treatment is due primarily to a COX2-independent mechanism based on the rapid activation (within minutes) of AMPK, most likely through Ca^2+^-dependent induction of the upstream kinase, CaMKK. The involvement of the latter kinase would be in partial agreement with Pyrko and colleagues [10], who demonstrated that celecoxib is able to induce Ca^2+^ release from endoplasmic reticulum (ER) stores. However, in contrast to Pyrko and colleagues, we were unable to detect activation of ER stress response following celecoxib treatment in LAMA-84 cells (data not shown).

Activation of AMPK seems to precede mTORC1 and mTORC2 inhibition, restoration of GSK3β activity, β-catenin down-regulation and the impairment of CML cell proliferation. Accordingly, partial knock-down of AMPKα expression is sufficient to restrain the molecular events described above as well as the anti-leukemic effect of celecoxib. In support of the central role of AMPK in the molecular and biological events triggered by celecoxib, other groups have demonstrated that activation of AMPK, in presence of functional p53, may activate the G1/S checkpoint, leading to replicative senescence of cancer cells [41]. In line with this observation, we demonstrated that celecoxib is able to increase the transcription of p16^INKa^, a prototypical marker of senescence [42].

Activation of AMPK would also explain the ability of celecoxib to impair at least mTORC1, since AMPK restores the inhibitory action of Rheb onto this protein complex. Moreover, inhibition of mTORC1, on its own, is likely to cause GSK3β re-activation due to the suppression of S6K kinase, which normally phosphorylates GSK3β [25,43]. Thus, it was not surprising that there was a similar decrease in colony formation of CML cells after treatment with celecoxib or the mTORC1 inhibitor rapamycin. However, co-treatment experiments revealed more effective inhibition of CML colony formation, suggesting non-redundant mechanisms of action of these two drugs. Indeed, celecoxib, but not rapamycin, impaired Akt phosphorylation, most likely via inhibition of mTORC2. Moreover, only celecoxib caused down-regulation of β-catenin, an event that is highly relevant for its anti-leukemic activity. In this regard, several groups have demonstrated that activation of the Wnt/β-catenin/Tcf-Lef pathway is essential for maintaining the self-renewal of leukemic stem cell (LSC) [22]. Moreover, it is becoming clear that aberrant activation of the β-catenin/Tcf-Lef axis promotes the transition of CML from chronic phase to blast crisis [19,21,22]. Indeed, BCR-ABL enables β-catenin-driven transcription independently of Wnt stimulation, since: i) it inhibits the GSK3β-dependent degradation of β-catenin; and ii) facilitates the interaction of β-catenin with Tcf/Lef [44]. Based on these observations, Heidel and colleagues [19] hypothesized that inhibition of canonical Wnt signalling would hamper CML resistance to imatinib, thereby maximizing the therapeutic benefit of a co-treatment with TKIs. In agreement with this concept, celecoxib reduced the clonogenic potential of TKI-resistant CML cells (including cell lines carrying the T315I BCR-ABL mutation) and synergized with imatinib upon co-treatment of TKI-responsive cells. Intriguingly, induction of β-catenin degradation by celecoxib has been previously reported in human colon carcinoma [45].

In favour of a possible therapeutic use of celecoxib in the management of CML patients, we found that, in contrast to what described for TKIs [46,47], this drug does not activate autophagy despite its ability to impair mTORC1 (data not shown); although this may seem contradictory, it is known that mTORC1 inhibition does not invariably induce the autophagic response, as already shown for rapamycin [48].

Even more translationally-relevant, celecoxib also suppressed proliferation and colony formation and induced apoptosis of CD34^+^ CML cells and primary Ph^+^ ALL cells, respectively; this effect appears to be relatively selective as it was significantly less pronounced in normal CD34^+^ progenitors from healthy donors.

The argument most commonly used against the specificity of COX2-independent, anti-proliferative, effects of celecoxib is that they were observed at drug concentrations higher than 100 μM. In this regard, most of our experiments were performed at a concentration of 10-25 μM and the colony assays demonstrated that celecoxib may be active even at lower concentrations, with an EC50 of 0.5 μM in the LAMA-84 cell line. These data are important since it was shown that, at least in mice, plasmatic concentrations as high as 45 μM are well tolerated *in vivo* [49]. Noteworthy, due to the lipophilic nature of celecoxib, this drug is concentrated three to four times more in the bone marrow than in plasma [49]; therefore, a concentration of 25 μM celecoxib in the hematopoietic compartment can be achieved at a dose of 950 mg, not too far from the daily dose commonly used in the treatment of FAP patients (800 mg, divided in two administrations).

We have shown that celecoxib is able to inhibit CML blast proliferation and survival, *in vitro* and in patient-derived cells, at therapeutically-relevant concentrations, through AMPK-dependent impairment of mTOR and β-catenin activity. This mechanism is COX2-independent and is mimicked by dimethyl-celecoxib and OSU03012, a drug currently being examined in clinical trials, but not by rofecoxib and indomethacin, two COX2-specific inhibitors. Given the good tolerability of celecoxib and OSU-03012, these findings support further investigations of these agents for the treatment of Ph^+^ hematological malignancies.

## MATERIALS AND METHODS

### In vitro cell cultures

K562, LAMA-84, JURL-MK1, HeLa were obtained from the American Type Culture Collection (ATCC, Rockville, MD, USA). BV173 CML-lymphoid blast crisis cell line and the imatinib-resistant cell lines BV173R (carrying the T315I mutation of BCR-ABL oncoprotein; [50]), KCL-22R (which gained imatinib resistance with a BCR-ABL-independent mechanism and through chromosomal aberrations; [51]), and K562R (which is imatinib-resistant through upregulation of Lyn kinase [52]), were kindly provided by Dr N.J. Donato (University of Michigan). Cells were grown according to ATCC instructions and kept in culture at 37 °C, under a 5% CO_2_ humidified atmosphere

The LAMA-84 cell line expressing the β-catenin S33Y mutant was obtained by gene transfection (see details in the text) and was maintained in a RPMI-1640-based medium, as described for the parental cell line.

AMPKα-silenced cells and their scramble-transduced counterpart, SCR, were obtained by lentiviral delivery of microRNAs (see further in the text), and were cultured as the parental LAMA-84 cell line.

### In vitro culture of Ph^+^ primary leukemia cells

Primary Ph^+^ ALL bone marrow cells were kindly provided by Dr Michael Caligiuri (Ohio State University, Columbus, OH) and Dr Martin Carroll (University of Pennsylvania, Philadelphia, PA). Cells were seeded on a substrate of adherent, mitomycin C-treated OP9 stromal cells and maintained in SFEM (Stem Cell Technology, Vancouver, Canada) supplemented with IL-3 (10 ng/ml), IL-7 (10 ng/ml), FLT3L (20 ng/ml) and SCF (30 ng/ml) (ProSpec, Israel).

CD34^+^ CML cells were kindly provided by Dr Tessa Holyoake (University of Glasgow, United Kingdom) and cultured in SFEM supplemented with IL-3 (20 ng/ml), IL-6 (20 ng/ml), SCF, and thrombopoietin (10 ng/ml).

Commercially purchased (Stem Cell Technologies) cord blood CD34^+^ cells were cultured in SFEM (Stem Cell Technologies) enriched with the CC100 cytokine cocktail (SCF, 100 ng/ml; FLT3L, 100 ng/ml; IL-3, 20 ng/ml; IL-6, 20 ng/ml).

Ph^+^ and normal CD34^+^ primary cells were kept in culture at 37 °C, under a 5% CO_2_ humidified atmosphere. Cell counts were performed using 0.4% Trypan Blue Solution and a Neubauer hemocytometer.

### Chemicals and antibodies

A list of chemicals used and their suppliers is provided in Supplementary Material. 2,5-dimethyl-celecoxib was synthesized by Prof Minassi according to published methods [53].

### 3-(4,5-Dimethylthiazol-2-Yl)-2,5-Diphenyltetrazolium Bromide (MTT) assay

MTT assays were performed according to standard procedures.

### Analysis of cell cycle distribution by flow cytometry

Propidium iodide staining of DNA content was performed as described [54] and cell cycle analysis was performed in an Accuri-C6 flow cytometer and analysed using ModFit LT software.

### Staining of apoptotic nuclei

Nuclear DNA integrity was assessed through microscopy of cells stained with the DNA-binding, supravital dye, Hoechst-33342 (Sigma-Aldrich Inc., Milan, Italy). *Caspase activation assay.* Activated Caspase 3-7 was detected with CellEvent Caspase 3-7 Green Detection Reagent (Life Technologies) after washing with PBS Ph^+^ ALL cells seeded on the feeder layer of OP9 stromal cells.

### Colony formation assays

Cells were treated, for 24 hours at a density of 1x10^5^cells/mL, with the concentrations indicated for each drug or with vehicle (0.1% DMSO). Next, 1,250 (or 10,000 for primary CML) cells/well were seeded into a semisolid culture media consisting of 80% methylcellulose (MethoCult, STEMCELL Tech., Milan, Italy) and 20% RPMI-1640 (fully supplemented) or in methylcellulose medium supplemented with a cytokine cocktail (CC100; Stem Cell Technologies). After 6-10 days, colonies were counted using a phase-contrast microscope.

### Immunoblottings

Whole-cell extracts were obtained using the RIPA lysis buffer (50 mM Tris-HCl pH 7.4; 150 mM NaCl, 0.5 mM EDTA pH 8, 1% Igepal, 0.1% SDS), supplemented with 1 mM phenylmethylsulphonyl fluoride, 1 mM sodium orthovanadate, 50 mM sodium fluoride, 10 mM glycerophosphate, 0.5 mM dithiothreitol and standard protease inhibitor cocktail (Roche Applied Science, Indianapolis, IN, USA). Lysates were clarified by centrifugation at 14,000g for 15 min at 4°C and equal amount of proteins (30 μg) were loaded on SDS-PAGE gels.

Antibodies were all prepared in TRIS-buffered saline solution containing with 0.1% Tween-20 (T-TBS), and supplemented with 3-5% non-fat dried milk or 3-5% bovine serum albumin, depending on the indications of the manufacturers.

### RNA extraction, reverse transcription and quantitative Real-Time PCR (qRT-PCR)

Total RNA was extracted from approximately 5 x10^6^ cells using TRI-Reagent (Sigma Aldrich Inc., Milan, Italy) and retrotrascribed using Im-Prom-II Reverse Transcriptase (Promega, WI, USA).

After setting gradient PCRs to verify primers specificity and melting temperature, standard curves of SYBR Green fluorescence were generated for each gene tested in order to evaluate primer efficiency.

qRT-PCRs were performed on a 96-well plate, in triplicate, and fluorescence intensity assessed using the CFX96 Real-Time PCR Detection Systems (Bio-Rad Inc., Milan, Italy). The following conditions were adopted: 12.5 μL Maxima SYBR Green/ROX qPCR Master Mix (Thermo Fisher Scientific Inc., Milan, Italy), 0.1 μM of forward and reverse primers, and 5 μL of 1:5 diluted cDNA, in a total volume of 25 μL/reaction. A list of the primers used (human *CTNNB1,* human *c-myc,* human *p16^INK4a^*,** human *GAPDH)* is presented in the Supplementary Section. Transcripts were normalized to the expression of glyceraldehyde-3-phosphate dehydrogenase (GAPDH) mRNAs. For each gene, the threshold cycle (*C_t_*) was calculated using CFX Manager Software (Bio-Rad Inc., Milan, Italy). The *C_t_* of treated cells was compared to the *C_t_* generated by the control cells and *C_t_* was calculated as the difference between *C_t_* values, determined using the equation 2^-Ct^.

### Plasmids, transfection, and lentiviral infections

LAMA-84 β-catenin S33Y cells were obtained by stable transfection of a pcDNA3 plasmid carrying the cDNA for a FLAG-tagged degradation-resistant mutant of β-catenin in which serine 33 has been replaced by a tyrosine (Addgene, Cambridge, MA, USA). Cells were selected for 10 days with G418 (0.8 mg/mL; Sigma Aldrich Inc., Milan, Italy). Expression of the exogenous protein (S33Y β-catenin) was verified by immunoblots with the anti-FLAG rabbit polyclonal antibody.

Lentiviral bicistronic vectors (pGIPZ) carrying the reporter gene turbo-Green Fluorescence Protein (tGFP) and non-targeting (SCR) or -specific miRNAs (all obtained through the Open Biosystems library of the University College of London Cancer Institute) were used to knock-down expression of AMPKα in LAMA-84 cells. Viruses were prepared as described in the supplementary section. The efficiency of nine different clones of AMPKα-specific miRNAs was assessed preliminarily through western blotting with anti-AMPKα rabbit polyclonal antibody (Cell Signaling Technology Inc., Danvers, MA, USA). Flow cytometry and cell sorting by means of green-fluorescence intensity (the FL1-A parameter, BioRad S3) were used to dissect high-expressing (top10% of the FL1-A channel, indicated as highGFP) from low expressing (bottom 10%, indicated as lowGFP) cells. HighGFP cells, having the least AMPKα expression, were routinely compared to lowGFP cells, which express AMPKα at levels comparable to wild-type LAMA-84 cells.

### Statistical analysis

The results in this study are presented as mean S.E.M of the results obtained from independent experiments. Statistical analyses were performed using Student's t-test and significance was indicated as follows: P<0.05 (*), P<0.01 (**), P<0.001 (***).

## SUPPLEMENTARY MATERIALS AND METHODS


