# Supplementary material for: Celecoxib inhibits proliferation and survival of chronic myelogeous leukemia (CML) cells via AMPK-dependent regulation of β-catenin and mTORC1/2
Source: Oncotarget. 2016 Nov 7;7(49):81555–70. doi: 10.18632/oncotarget.13146 (PMC5348412; doi:10.18632/oncotarget.13146)
Supplement: Supplementary file 1 [file oncotarget-07-81555-s001.pdf]

Celecoxib inhibits proliferation and survival of chronic myelogeous leukemia (CML) cells via AMPK-dependent regulation of β-catenin and mTORC1/2

SUPPLEMENTARY MATERIALS AND METHODS

Chemicals

Celecoxib (4-[5-(4-methylphenyl)-3-(trifluoromethyl)-1H-pyrazol-1-yl]benzenesulfonamide, 200 mM stock solution; Chemos GmbH, Regenstauf, Germany); imatinib (IUPAC name: 4-[(4-methylpiperazin-1-yl)methyl]-N-[4-methyl-3-[(4-pyridin-3-ylpyrimidin-2-yl)amino]phenyl] benzamide; 50 mM stock solution), LKT Laboratories Inc., St. Paul, MN, USA; metformin (1,1-dimethylbiguanide hydrochloride, 200 mM stock solution; Selleckchem GmbH, Munich, Germany); rofecoxib (3-(4-methylsulfonylphenyl)-4-phenyl-2H-furan-5-one, 50 mM stock solution; MSD Inc., Milan, Italy); MG132 (Z-Leu-Leu-Leu, 50 mM stock solution), BIO (10 mM stock solution), STO-609 (10 mg/mL stock solution), all purchased from Sigma-Aldrich Inc., Milan, Italy; these compounds, with the exception of metformin (reconstituted in milliQ water), were dissolved in 100% dimethyl sulfoxide (DMSO) and stored at +4 or -20 °C, according to manufacturers' specifications. Working concentrations of these compounds were freshly prepared for each experiment by diluting DMSO to 0.1% in milliQ (MilliPore) water.

Antibodies

The following primary antibodies were used: anti β-actin (clone AC-15 Sigma-Aldrich Inc., Milan, Italy); anti-COX1 (clone H-62, Santa Cruz Biotechnology Inc., Santa Cruz, CA, USA); anti-COX2 (clone C-20, Santa Cruz Biotechnology Inc., Santa Cruz, CA, USA); anti-c-Myc (clone 9E10, Santa Cruz Biotechnology Inc., Santa Cruz, CA, USA); anti-β-catenin (clone 14, BD Bioscience, Milan, Italy); anti-active β-catenin (non-phospho ser33/37/thr41, Cell Signaling Technology Inc., Danvers, MA, USA); anti-phospho-AMPKα (thr172 clone 40H9) and AMPK-α (Cell Signaling Technology Inc., Danvers, MA, USA); anti-phospho-GSK-3β (ser9) (abcam Plc., Cambridge, UK) and anti-GSK-3β (Cell Signaling Technology Inc., Danvers, MA, USA); anti-phospho-Akt (thr308) (abcam Plc., Cambridge, UK); anti-phospho-Akt (ser473-D9E) and anti-pan-Akt (Cell Signaling Technology Inc., Danvers, MA, USA); anti-phospho-mTOR (Ser2448), anti-phospho-mTOR (ser2481) and anti-pan-mTOR (clone 7C10) (Cell Signaling Technology Inc., Danvers, MA, USA); anti-phospho-p70 S6 Kinase (thr389) and anti-p70 S6 Kinase (Cell Signaling Technology Inc., Danvers, MA, USA); anti-phospho-4E-BP1 (thr37/46) and anti-4E-BP1 (Cell Signaling Technology Inc., Danvers, MA, USA).

RT-PCR

After an initial denaturation step at 95°C (10 min), each primer set was used (through 40 cycles of amplification) as follows: human *CTNNB1*, 5'-ACCAGCGCCGTACGTCCAT-3' forward and 3'-GC TAGGATGTGAAGGGCTCCG-5' reverse (104-bp amplicon), 60°C annealing temperature; human *c-myc*, 5'-CAAAGACAGCGGCAGCCC-3' forward and 3'-GCG AGGCGCAGGACTTG-5' reverse (164-bp amplicon), 64.4°C annealing temperature; human $p16^{INK4a}$, 5'-CAACCTGGGGCGACTTCAG-3' forward and 3'-GA CCAGCCAGCCCCTCC-5' reverse (201-bp amplicon); 61.7°C annealing temperature.

GAPD was assessed using the 5'-CAA GGTCATCCATGACAACTTTG-3' forward and the 3'-GGGCAATCCACAGTCTTCTG-5' reverse primer (90-bp amplicon), 60°C annealing temperature.

Lentivirus packaging and infections

Lentiviral supernatants were prepared, according to manufacturer's instructions (GE-Dharmacon, UK), using HEK-293T cell lines for virus packaging. Cells were transfected with a mix (4:3:1) of pGIPZ, psPAX2 (carrying the HIV-derived *gag/pol*, *rev* and *tat* sequences) and pMD2.G plasmids (coding for the *env* protein of the *Vesicular Stomatitis Virus G*, required for virion pseudotyping) using the METAFECTENE® PRO reagent (Biontex Laboratories GmbH, Germany). Lentiviral particles were concentrated after two steps of centrifugation (1500 g, 4°C) in a solution containing polyethylene glycol (PEG MW 8000, Sigma Aldrich Inc., Milan, Italy) and then re-suspended in PBS (1/50 of the initial volume).

Titration was performed on HeLa cells infected with serial dilutions (1:5) of the viral suspension. Infection efficiency was calculated by assessing, through flow cytometry, percentages of cells expressing the tGFP reporter gene contained in the pGIPZ plasmid.

Infections were carried out by *spinoculation* procedure. Briefly, $1x10^6$ cells/mL, plated in 6-well

plates, were exposed to viruses at a concentration of 10 multiplicity of infection (MOI) in presence of 8 µg/mL of Polybrene® (Sigma Aldrich Inc., Milan, Italy). Incubation with viruses was allowed upon centrifugation for 45 min at 1,260 rpm; next, cells were incubated for 5 hours at 37°C before being switched to regular medium.

SUPPLEMENTARY FIGURES

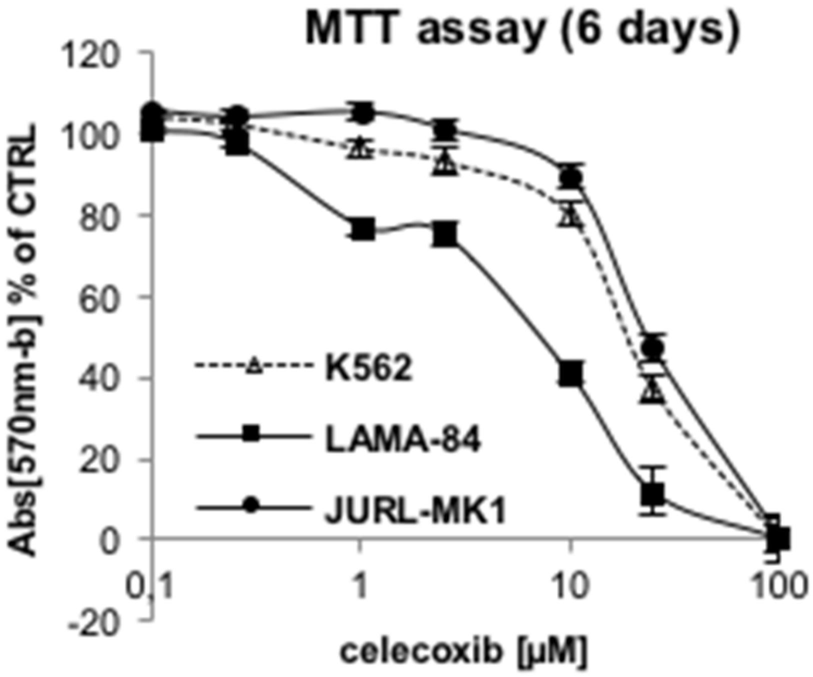

Supplementary Figure S1: Cell viability (MTT assay) of LAMA-84, JURL-MK1 and K562 cell lines treated for six days with increasing concentrations of celecoxib (0.1-100 µM). Data are presented as percent reduction of MTT absorbance of 0.1% DMSO-treated cells (CTRL) and represents averages of three independent experiments (± S.E.M.).

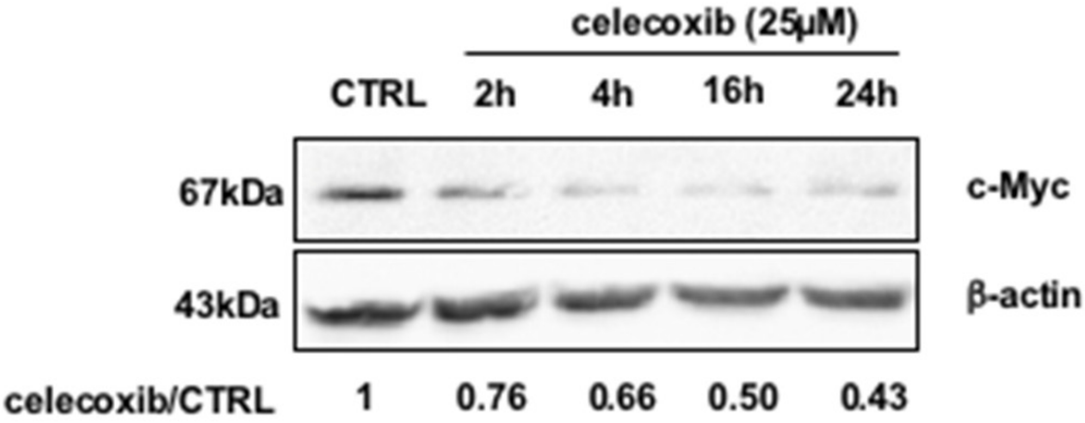

Supplementary Figure S2: c-Myc protein expression in celecoxib-treated LAMA-84 cells. Western blot shows c-Myc expression in celecoxib-treated LAMA-84 cells. Numbers underneath lanes represent the treated/control ratio of the optical densities of c-Myc expression normalized to β-actin levels.

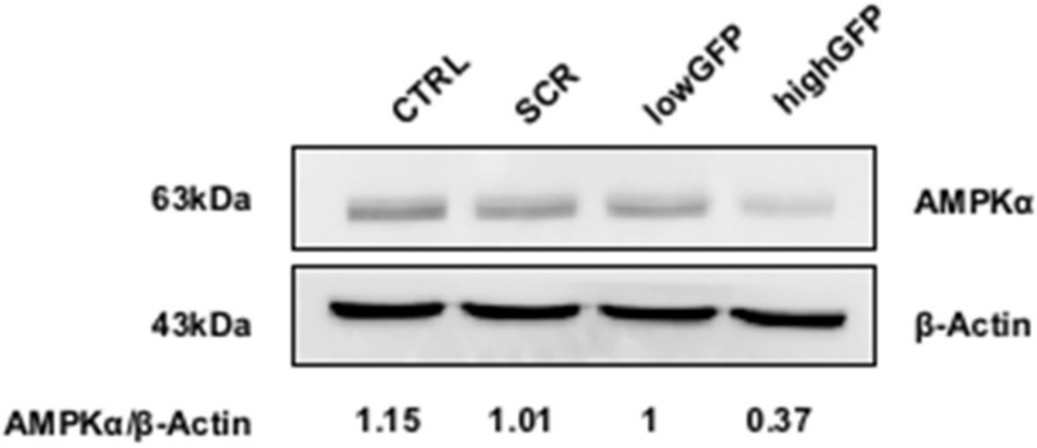

Supplementary Figure S3: AMPKα protein levels of parental (CTRL), *scramble*-transduced (SCR), and AMPKi-transduced LAMA-84 cells. Immunoblots of lowGFP (basal levels of AMPKα) and highGFP (low levels of AMPKα) LAMA-84 cells (see methods for explanations). Numbers displayed underneath lanes represent the ratio of the intensity of the AMPKα immune-reactive band over that of β-actin (used for loading normalization).

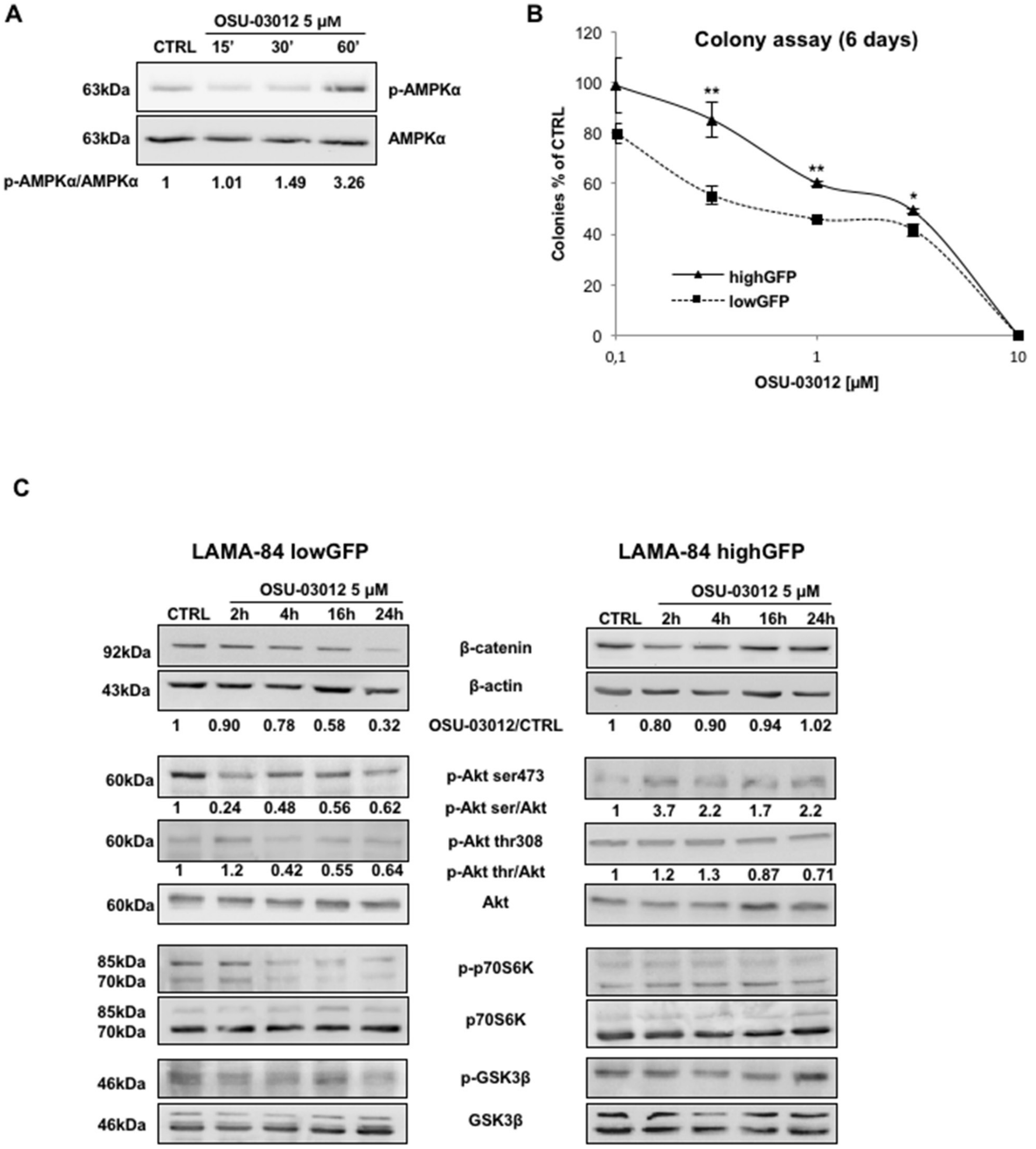

Supplementary Figure S4: Activation of AMP-dependent kinase (AMPK) contributes to the anti-leukemia effect of OSU-03012. A. Phosphorylation of AMPK in LAMA-84 cells treated with OSU-03012. Cells were exposed for 15 to 60 min to 5 µM OSU-03012. Protein lysates were probed with antibodies that recognize the α-subunit of AMPK (AMPKα) or its active form, phosphorylated on thr-172 (p-AMPKα). Values underneath lanes indicate the ratio of the optical densities of p-AMPKα immuno-reactive bands over those of total AMPKα. **B.** Colony assay of lowGFP- (basal levels of AMPK) and highGFP (low levels of AMPK) LAMA-84 cell lines (see methods for an explanation) treated with increasing concentrations of OSU-03012 (0.1-10 µM). Cells (1,250/well) were grown for 6 days on 80% methylcellulose culture media after treatment with OSU-03012. Results are expressed as percentages of colonies in drug-treated groups as compared to 0.1% DMSO-treated controls (CTRL). Values represent the mean of three independent experiments made in duplicate (± S.E.M.). $^{*}P \leq 0.05$, $^{**}P \leq 0.01$, $^{***}P \leq 0.001$ **C.** Analysis of OSU-03012-regulated signalling pathways in lowGFP (left panel) and highGFP (right panel) LAMA-84 cell lines. Cells were treated with 5 µM OSU-03012 for 2 to 24 hours before preparation of protein lysates. Immunoblots were performed by using primary antibodies specific for: β-catenin (**β-cat**); p70S6 kinase (**p70S6K**) or its active form, phosphorylated on thr-389 (**p-p70S6K**); GSK3β or its inactive form phosphorylated on ser-9 (**p-GSK-3b**); Akt or its active forms phosphorylated either on ser-473 (**p-Akt ser473**) or thr-308 (**p-Akt thr308**). Optical densities of β-catenin immuno-reactive bands were normalised by the intensity of β-actin in order to calculate treatment-to-control (**OSU-03012/CTRL**) ratios. Controls were obtained by treating cells with 0.1 % DMSO. Optical densities of total p70S6K, GSK3β and Akt were assessed and used to calculate ratios to their phosphorylated counterparts. All values are displayed underneath.

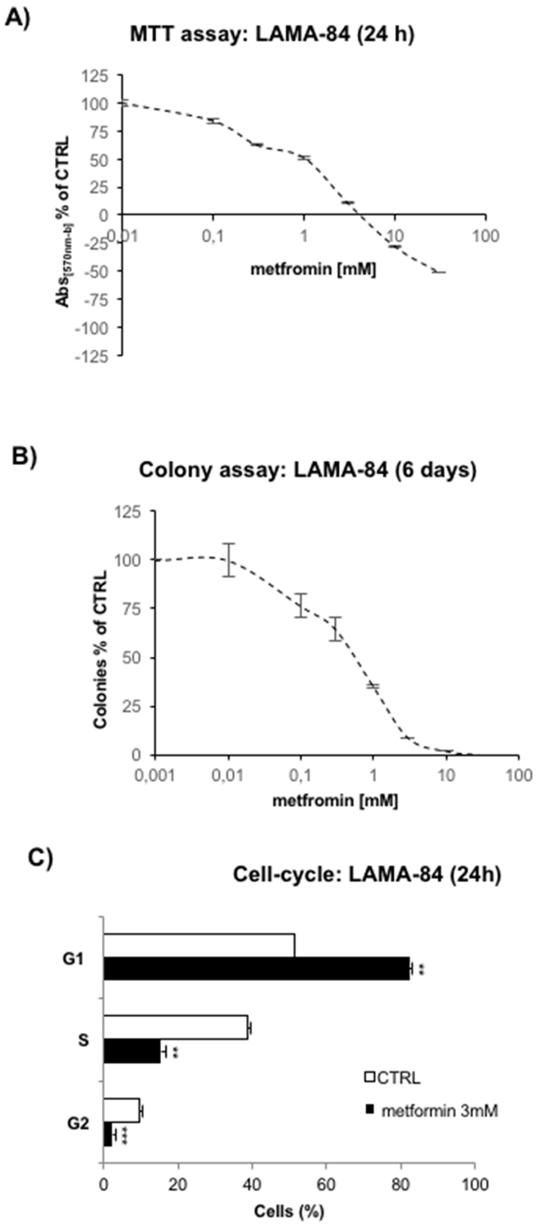

	G1	S	G2
CTRL	51.4±0.15	38.9±0.69	9.7±0.81
metformin (3 mM)	82.3±0.90	15.5±1.38	2.2±0.91

Supplementary Figure S5: Effect of metformin on viability, colony formation and cell cycling of LAMA-84 cells. A. Cell viability (MTT assay) of LAMA-84 treated for 24 h with increasing concentrations of metformin (0.01-30 mM). Data are presented according to NCI screening methodology (see legend for Figure 1) and represents averages of three independent experiments (± S.E.M.). *$^*P \leq 0.05$, $^{**}P \leq 0.01$, $^{***}P \leq 0.001$* **B.** Colony assay of LAMA-84 cells treated with increasing concentrations of metformin (0.01-30 mM). Cells (1,250/well) were grown for 6 days on 80% methylcellulose culture media after treatment with metformin. Results are expressed as percentages of colonies in drug-treated groups as compared to 0.1% DMSO-treated controls (CTRL). Values represent the mean of three independent experiments made in duplicate (± S.E.M.). *$^*P \leq 0.05$, $^{**}P \leq 0.01$, $^{***}P \leq 0.001$* **C.** LAMA-84 cell-cycle distribution assessed by flow cytometry after propidium iodide staining and treatment with 3 mM metformin. Results (means ± S.E.M of n=3 samples; *$^*P \leq 0.05$, $^{**}P \leq 0.01$, $^{***}P \leq 0.001$*) depict a representative experiment of three experiments that yielded overlapping results.

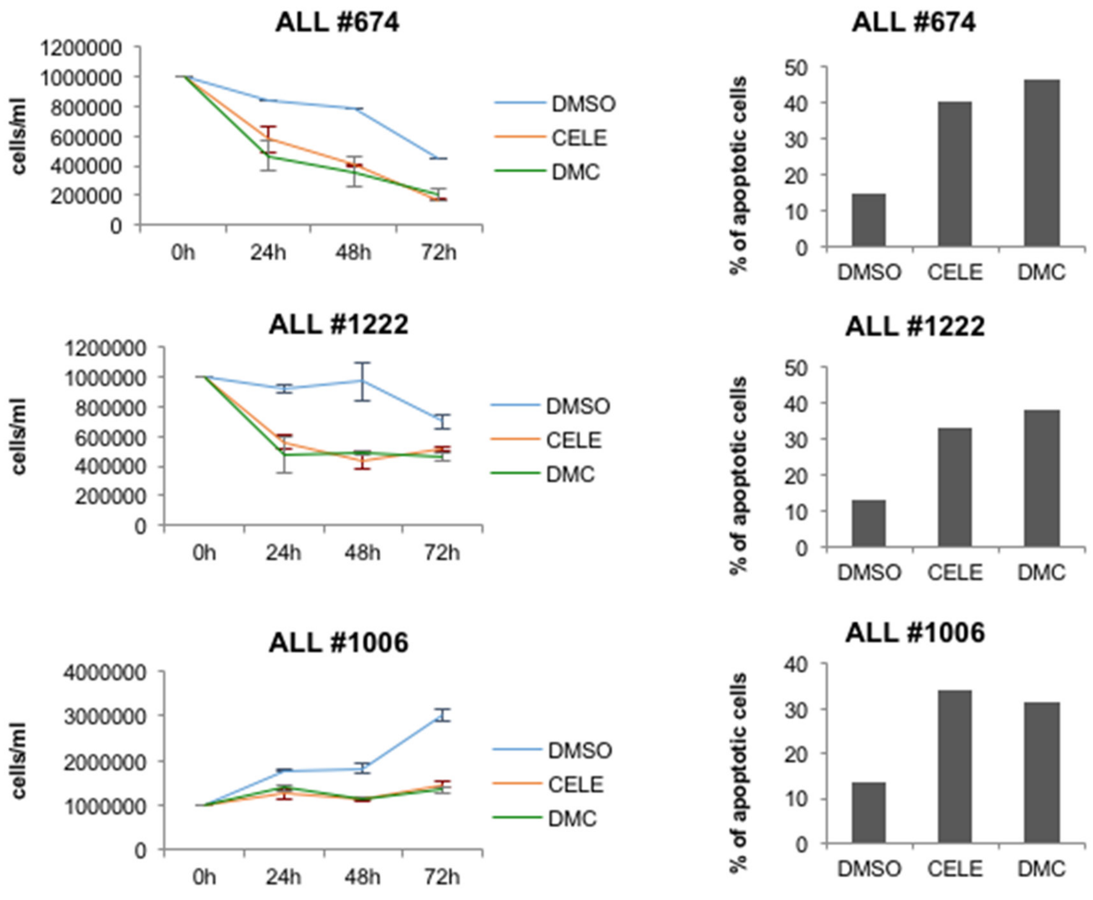

Supplementary Figure S6: Effect of celecoxib or dimethyl-celecoxib on proliferation and survival of Ph⁺ primary ALL cells. Left, Cell counts of celecoxib or dimethyl-celecoxib (DMC) treated Ph⁺ primary ALL cells. Cells were seeded at 100,000 cells/ml and treated with DMSO (0.1%) or 25 µM each of celecoxib or DMC. Cell counts were performed at 24, 48 and 72 hours; **Right,** % apoptosis (active caspase 3/6) in drug-treated Ph⁺ primary ALL cells.
